# Invasive cattle ticks in East Africa: morphological and molecular confirmation of the presence of *Rhipicephalus microplus* in south-eastern Uganda

**DOI:** 10.1186/s13071-020-04043-z

**Published:** 2020-04-03

**Authors:** Dennis Muhanguzi, Joseph Byaruhanga, Wilson Amanyire, Christian Ndekezi, Sylvester Ochwo, Joseph Nkamwesiga, Frank Norbert Mwiine, Robert Tweyongyere, Josephus Fourie, Maxime Madder, Theo Schetters, Ivan Horak, Nick Juleff, Frans Jongejan

**Affiliations:** 1 College of Veterinary Medicine Animal Resources and Biosecurity, Makerere University, P.O. Box 7062, Kampala, Uganda; 2 ClinVet International (Pty) Ltd, P.O. Box 11186, Universitas, 9321 Bloemfontein South Africa; 3 Clinglobal, B03/04, The Tamarin Commercial Hub, Jacaranda Avenue, Tamarin, 90903 Mauritius; 4 Department of Veterinary Tropical Diseases, Faculty of Veterinary Science, University of Pretoria, Private Bag X04, Onderstepoort, 0110 South Africa; 5 Bill & Melinda Gates Foundation, Seattle, WA USA; 6 Utrecht Centre for Tick-borne Diseases (UCTD), Faculty of Veterinary Medicine, Utrecht University, Yalelaan 1, 3584 CL Utrecht, The Netherlands

**Keywords:** *Rhipicephalus microplus*, Ticks, Serere district, Uganda, Tick-borne diseases

## Abstract

**Background:**

*Rhipicephalus microplus*, an invasive tick species of Asian origin and the main vector of *Babesia* species, is considered one of the most widespread ectoparasites of livestock. The tick has spread from its native habitats on translocated livestock to large parts of the tropical world, where it has replaced some of the local populations of *Rhipicephalus decoloratus* ticks. Although the tick was reported in Uganda 70 years ago, it has not been found in any subsequent surveys. This study was carried out to update the national tick species distribution on livestock in Uganda as a basis for tick and tick-borne disease control, with particular reference to *R. microplus*.

**Methods:**

The study was carried out in Kadungulu, Serere district, south-eastern Uganda, which is dominated by small scale livestock producers. All the ticks collected from 240 cattle from six villages were identified microscopically. Five *R. microplus* specimens were further processed for phylogenetic analysis and species confirmation.

**Results:**

The predominant tick species found on cattle was *Rhipicephalus appendiculatus* (86.9 %; *n* = 16,509). Other species found were *Amblyomma variegatum* (7.2 %; *n* = 1377), *Rhipicephalus evertsi* (2.3 %; *n* = 434*)* and *R. microplus* (3.6 %; *n* = 687). Phylogenetic analysis of the *12S* rRNA, *16S* rRNA and ITS2 gene sequences of *R. microplus* confirmed the morphological identification.

**Conclusions:**

It is concluded that *R. microplus* has replaced *R. decoloratus* in the sampled villages in Kadungulu sub-county, since the latter was not any longer found in this area. There is currently no livestock movement policy in force in Uganda, which could possibly limit the further spread of *R. microplus* ticks. Future surveys, but also retrospective surveys of museum specimens, will reveal the extent of distribution of *R. microplus* in Uganda and also for how long this tick has been present on livestock without being noticed.
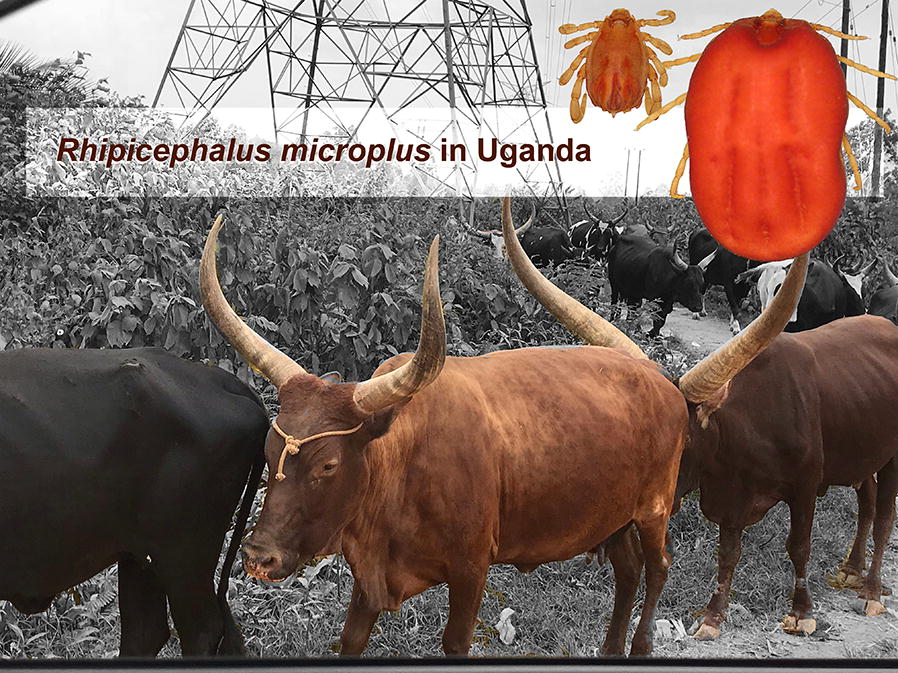

## Background

The Asian blue tick, *Rhipicephalus (Boophilus) microplus* (Canestrini, 1888), is one of the most important tick species infesting livestock in many parts of the world [1]. *Rhipicephalus microplus* has extended its distribution through the translocation of tick-infested cattle. In some regions in Africa, *R. microplus* has successfully competed and replaced the close related African blue tick, *Rhipicephalus decoloratus* [2–4]. *Rhipicephalus microplus* is vector of *Babesia bovis* and *Babesia bigemina* causing extensive production losses [5–7].

*Rhipicephalus microplus* has been introduced from Asia on cattle exported to East and South Africa *via* Madagascar [3]. Similarly, *R. microplus* was introduced into Ivory Coast and Benin from Brazil 10 years ago [8]. Since then, it has spread to Burkina Faso, Mali, Togo and very recently into Nigeria and Cameroon [3, 8–12]. This species is now well established in the southern and eastern fringes of South Africa [2, 7]. Displacement of local *R. decoloratus* populations in the countries where *R. microplus* was introduced could have resulted from the faster life-cycle of *R. microplus*, sterile off-spring of interspecific mating [2] or because of the higher degree of acaricide resistance of *R. microplus* [2].

There are a few isolated reports of *R. microplus* in East Africa, notably Tanzania and South Sudan [13, 14]. Some of these reports are over 30 years old [15, 16]; hence, this tick species could have spread to several parts of East Africa. Given that *R. microplus* is an invasive tick species, such isolated reports are likely to be due to a lack of regional or country-wide tick surveys and the distribution may be wider than reported. Furthermore, the differential diagnosis of *R. microplus* and *R. decoloratus* in East Africa and *R. decoloratus*, *Rhipicephalus annulatus* and *Rhipicephalus geigyi* in West Africa is difficult because of similarities in morphology and their small size [4].

There are no up-to-date Ugandan or East African tick surveys. As a result, despite records of *R. microplus* 70 years ago in the Uganda [17], this has never been confirmed. This study was carried out in one of the high tick density districts of south-eastern Uganda to update national tick species data as a basis for a national tick and tick-borne disease control strategy.

## Methods

### Study area

**Fig. 1:**
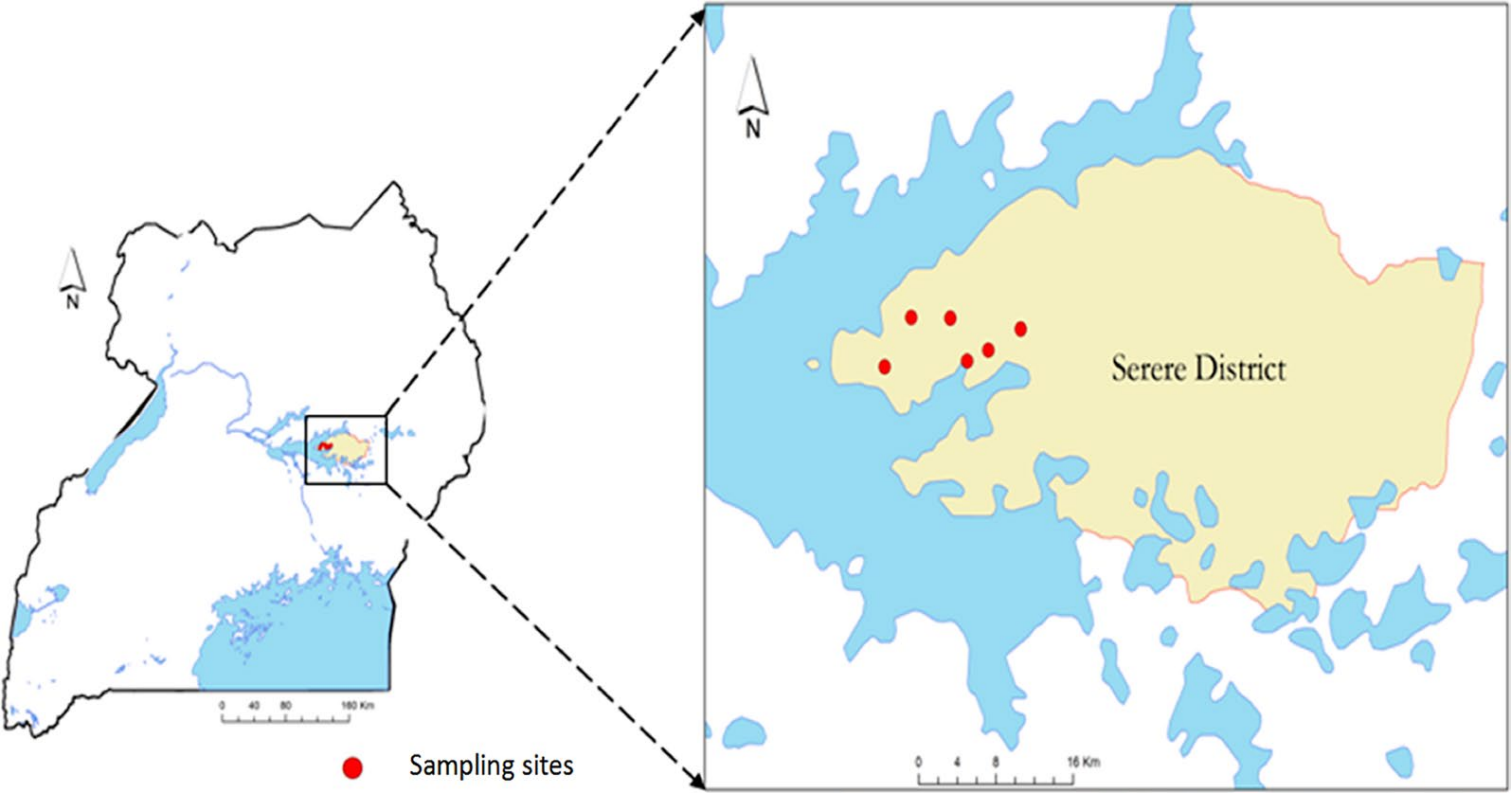
Study area: Serere district, south-eastern Uganda. Red dots indicate the locations of the 6 villages from which 240 cattle were randomly selected and included in this study

The study was carried out in Serere district, south-eastern Uganda, in 2017. The district is made up of two rural counties (Kasilo and Serere), eight sub-counties (Bugondo, Kadungulu, Pingire, Labor, Atiira, Kateta, Chere and Serere/Olio) encompassing 254 cattle-owning villages. Tick collections were conducted in Kadungulu sub-county. Serere district was selected because it has a large number of small scale livestock producers (1–50 cattle per herd) whose potential to commercialise livestock production is primarily constrained by ticks and tick-borne diseases. Six of the 254 villages were randomly selected for this study (Fig. 1).

### Cattle herd and individual animal selection

Farmers in Serere district predominantly keep short-horn East-African Zebu cattle in communal village herds. Given that any animal sampled from each of these villages is likely to be infested with ticks, the number of villages (*n* = 6) and animals selected (*n* = 240) need not to be based on any rigorous statistical methods. However, to estimate the chance of finding *R. decoloratus* ticks, we conducted a power calculation assuming a prevalence of 5% at *P* = 0.01; we expected to find 18 *R. decoloratus* ticks in a sample size of 18,000 ticks [18]. In this study we sampled 19,007 ticks. Cattle were included in this study if they had not been sprayed against ticks for the past two weeks, were young (1–2 years old) and non-fractious. Young non-fractious animals were preferred for inclusion because they were easier to restrain and pose very low risk of injury to themselves or personnel. An average of 40 cattle was sampled from each of the six selected villages. All cattle sampling sites were geo-referenced prior to tick collection.

### Tick collection and identification

Selected cattle were physically restrained before half-body tick collections were carried out. Each of the collected ticks was morphologically identified to the genus level before they were preserved in 70% ethanol. The tick samples were then transferred to Makerere University for further species identification using taxonomic keys [19]. Five representative *R. microplus* specimens were selected for molecular species confirmation based on *12S* ribosomal RNA (*12S* rRNA), *16S* ribosomal RNA (*16S* rRNA) and the internal transcribed spacer 2 (ITS2) gene sequences [4, 20]. A taxonomically and molecularly confirmed *R. microplus* specimen was photographed under a stereomicroscope (Olympus model SZX7, Tokyo, Japan).

### DNA extraction

Prior to tick DNA extraction, each tick was cleaned in five one-minute steps, each step involving centrifugation at 10,000× *rpm* in freshly prepared 1.5 ml of phosphate-buffered saline (PBS). Individual clean ticks were immersed under liquid nitrogen for 5 min and thereafter crushed with a sterile mortar and pestle to create a tick homogenate. DNA was then extracted from each tick homogenate using DNeasy Blood and Tissue Kit (Qiagen, Germantown, MD, USA) following the manufacturers’ instructions. The presence and quality of DNA were checked by resolving 5 µl of the extracted DNA on a 1% agarose gel and viewing them under an ultraviolet transilluminator (Wagtech International, Thatcham, UK). The remaining DNA was stored at − 20 °C until use in the downward amplification steps.

### DNA amplification

PCR amplification was performed on *12S* rRNA and *16S* rRNA genes and the ITS 2 spacer using primers (Table 1) and thermocycling conditions as previously described [4, 19]. Each reaction was prepared into a final volume of 50 µl containing; 1×-reaction buffer (670 mM Tris-HC, pH 8.8, 166 μM (NH_4_)_2_SO_4_, 4.5 % Triton X-100, 2 mg/ml gelatin) (Bioline, Humber Road, London, UK), 0.25 mM of each dNTP, 0.25 mM each of forward and reverse primers, 1.56 U *BioTaq* DNA polymerase (Bioline, London, UK), 1.25 mM MgCl_2_, 32.2 µl of PCR grade water and finally 5 µl of the template DNA.

**Table 1:** List of primer sets used in PCR amplification of *12S* rRNA and *16S* rRNA genes and the ITS2 region [4, 20]

Primer name	Primer sequence (5’-3’)
16S-F	TTAAATTGCTGTRGTATT
16S-R1	CCGGTCTGAACTCASAWC
ITS2-F	ACATTGCGGCCTTGGGTCTT
ITS2-R	TCGCCTGATCTGAGGTCGAC
T1B	AAACTAGGATTAGATACCCT
T2A	AATGAGAGCGACGGGCGATGT

The *16S* ribosomal RNA gene was amplified in a thermocycler (Personal Thermocycler, Biometra, Göttingen, Germany) with initial denaturation of 94 °C for 5 min followed by 30 cycles at 94 °C for 30 s, 48 °C for 45 s, 72 °C for 45 s and a final extension at 72 °C for 7 min. Amplification of the ITS2 and *12S* ribosomal RNA was performed using similar thermocycling conditions to those of *16S* at annealing temperature of 55 °C and 52 °C, respectively. PCR products were resolved on 2% agarose gels. The resultant PCR products were sized against a 1 kb DNA molecular ladder (Bioline, London, UK). The expected PCR product sizes ranged between 300–1200 bp. PCR products were purified using QIAquick PCR Purification Kit (Qiagen, Germantown, MD, USA) and commercially Sanger-sequenced (Inqaba Biotec, Muckleneuk, Pretoria, South Africa).

### Gene sequence analysis

Each of the *12S* rRNA, *16S* rRNA and ITS2 tick sequences from this study were queried in a BLASTn search with default settings (NCBI BLASTn software version 2.6.10) [21] to reveal their identity. The query sequence identity was assigned/matched based on the hits (tick species sequences returned) with the highest identity scores (≥ 80%) and most significant E-values (closest to 0.0). The identified query sequences from this study were annotated and submitted to the GenBank database under the accession numbers MK332390, MK332391, KY688455, KY688459, KY688461 and KY688467.

Annotated sequences from this study were each analysed in a multiple sequence alignment (MSA) with their corresponding reference gene sequences downloaded from GenBank using the ClustalW algorithm in MEGA version 10 [22]. The MSA files were used to infer nucleotide similarity between sequences from this study and their corresponding nucleotide reference sequences from GenBank. Each of the data sequence sets were analysed in MSA using the ClustalW algorithm and trimmed in MEGA software version 10 [23, 24]. Phylogenetic analysis for each nucleotide sequence set was performed using the maximum likelihood method utilising the Tamura 3-parameter with Gamma distribution with 1000 bootstrap replicates [23] as the best-fit model to infer phylogenetic relatedness among the gene sets.

To evaluate the *12S* rRNA, *16S* rRNA and ITS2 sequence divergence of newly typed Ugandan *R. microplus* ticks and their corresponding reference sequences from GenBank, pairwise genetic distances were calculated in MEGA software version 10 [23] using default settings for each sequence.

## Results

### Tick collections

Adult ticks (*n* = 19,007) were collected upon completion of half-body counts from 240 cattle. The majority (86.9 %; *n* = 16,509) of these ticks were *Rhipicephalus appendiculatus*. Other tick species identified were *Amblyomma variegatum* (7.2 %; *n* = 1377), *R. microplus* (3.6 %; *n* = 687) and *Rhipicephalus evertsi* (2.3 %; *n* = 434*)*. The mean adult tick density was 79 ticks per animal. On average, the numbers of adult *R. appendiculatus*, *A. variegatum, R. microplus* and *R. evertsi* per animal were 138, 11, 6 and 4, respectively.

**Fig. 2:**
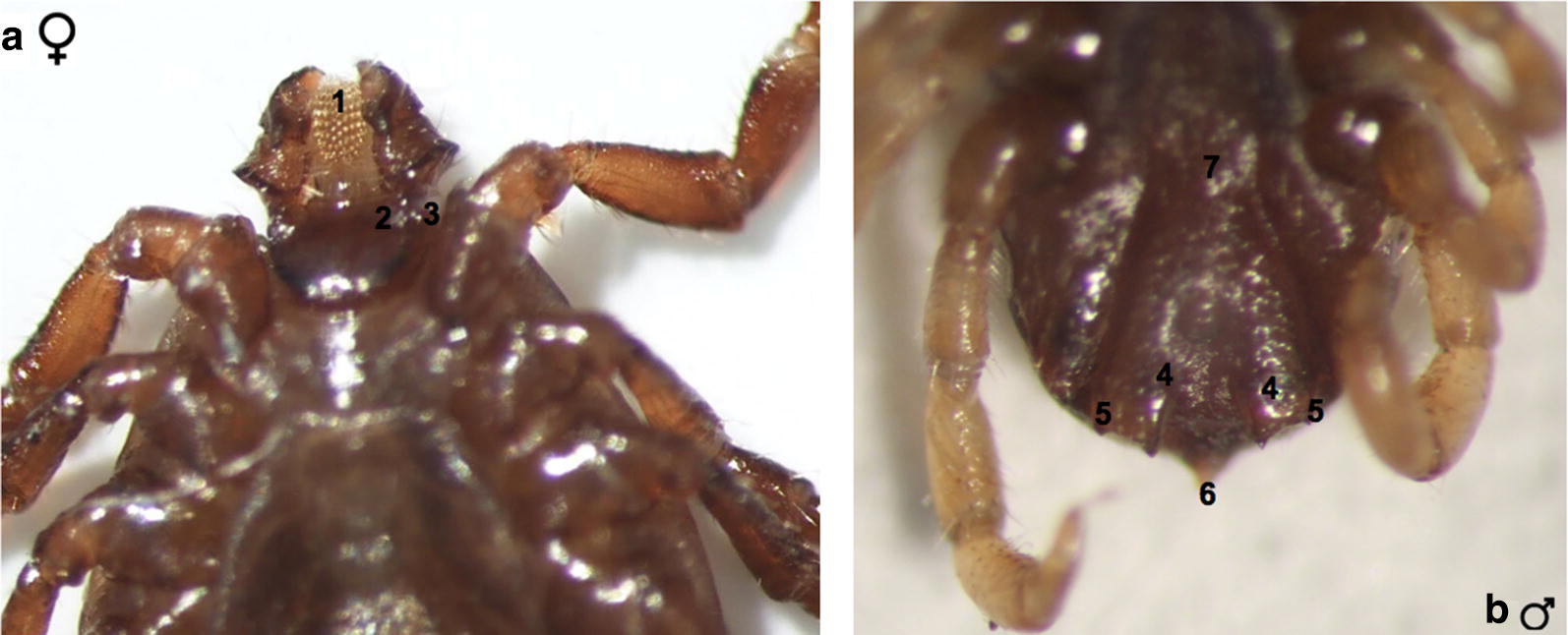
Morphological identification of *R. microplus*. **a** Female, ventral view (1, hypostomal teeth in a typical 4 + 4 column arrangement; 2, short palp of internal margin article 1 (lacks protuberance and is distinctly concave); 3, cornua). **b** Male, ventral view (4, distinctly small adenal plates; 5, ventral plate spurs (small accessory adanal plates); 6, caudal appendage; 7, genital aperture with a broad U-shape)

A taxonomically and molecularly confirmed *R. microplus* specimen was photographed under a stereomicroscope as shown in Fig. 2. Female *R. microplus* was characterized by hypostome teeth in a typical 4 + 4 column arrangement and internal margin palpal article 1 lacking protuberance and distinctly concave. Male *R. microplus* carried typical indistinct spurs on the ventral plates.

### Molecular confirmation of *R. microplus*

**Fig. 3:**
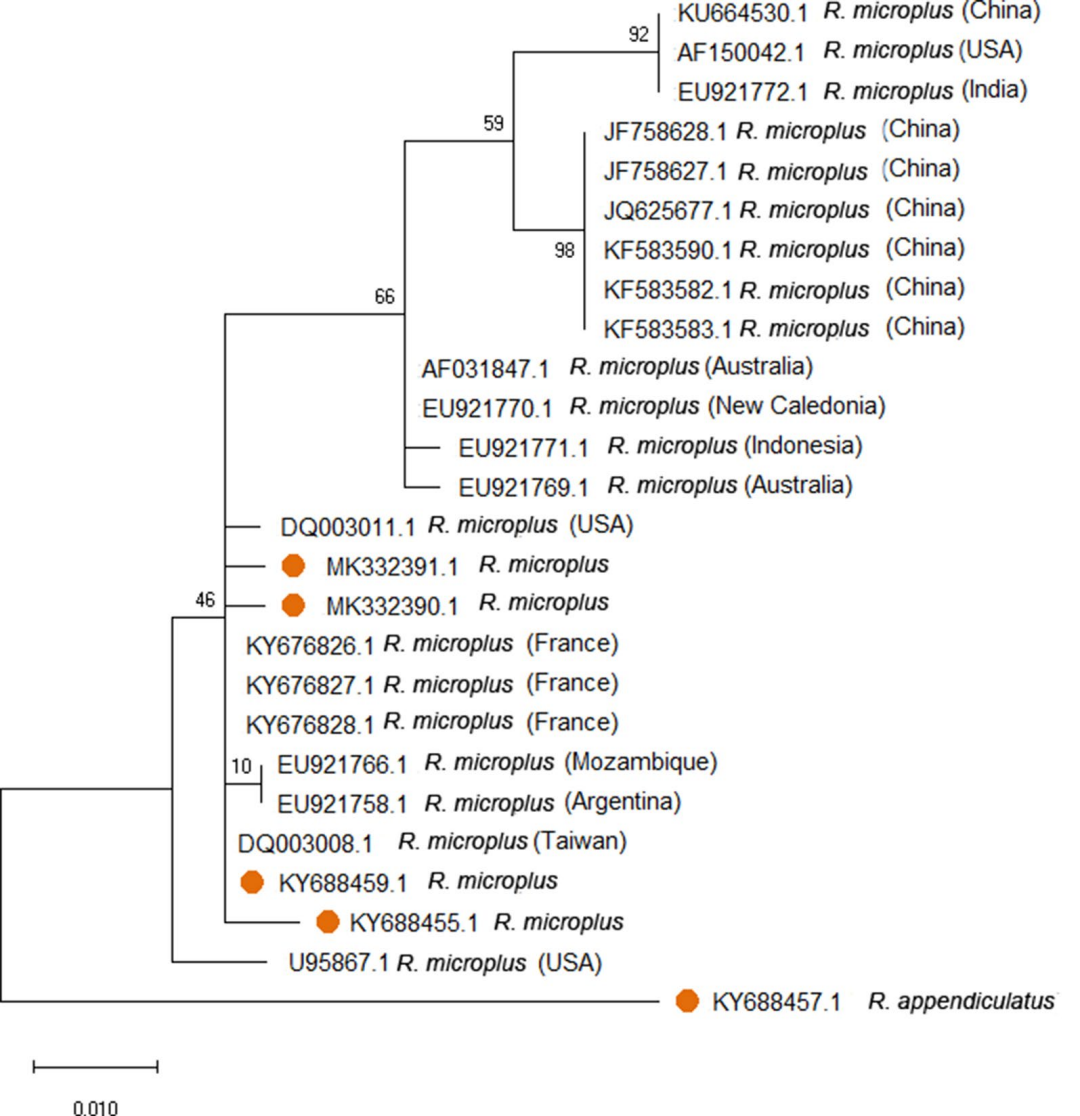
Phylogenetic analysis based on the tick *12S* ribosomal RNA gene. A phylogenetic tree based on *12S* rDNA sequences. The tree was generated by the maximum likelihood method based on the Tamura 3-parameter model. The analysis involved 26 nucleotide sequences. Orange circles represent samples sequenced in this study

**Fig. 4:**
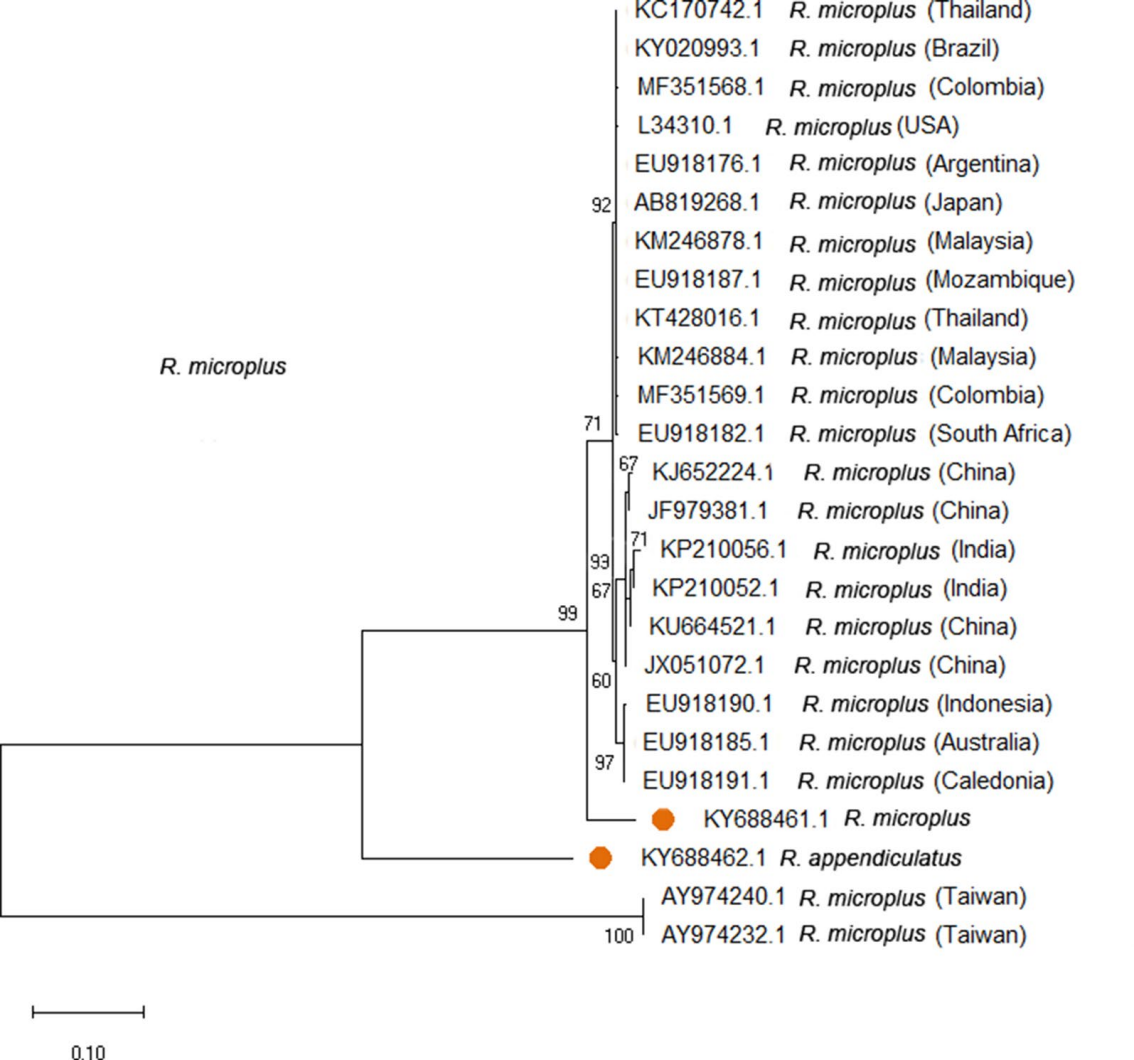
Phylogenetic analysis based on the tick *16S* ribosomal RNA gene. A phylogenetic tree based on *16S* rDNA sequences. The tree was generated by the maximum likelihood method based on the Tamura 3-parameter model. The analysis involved 25 nucleotide sequences. Orange circles represent samples sequenced in this study

**Fig. 5:**
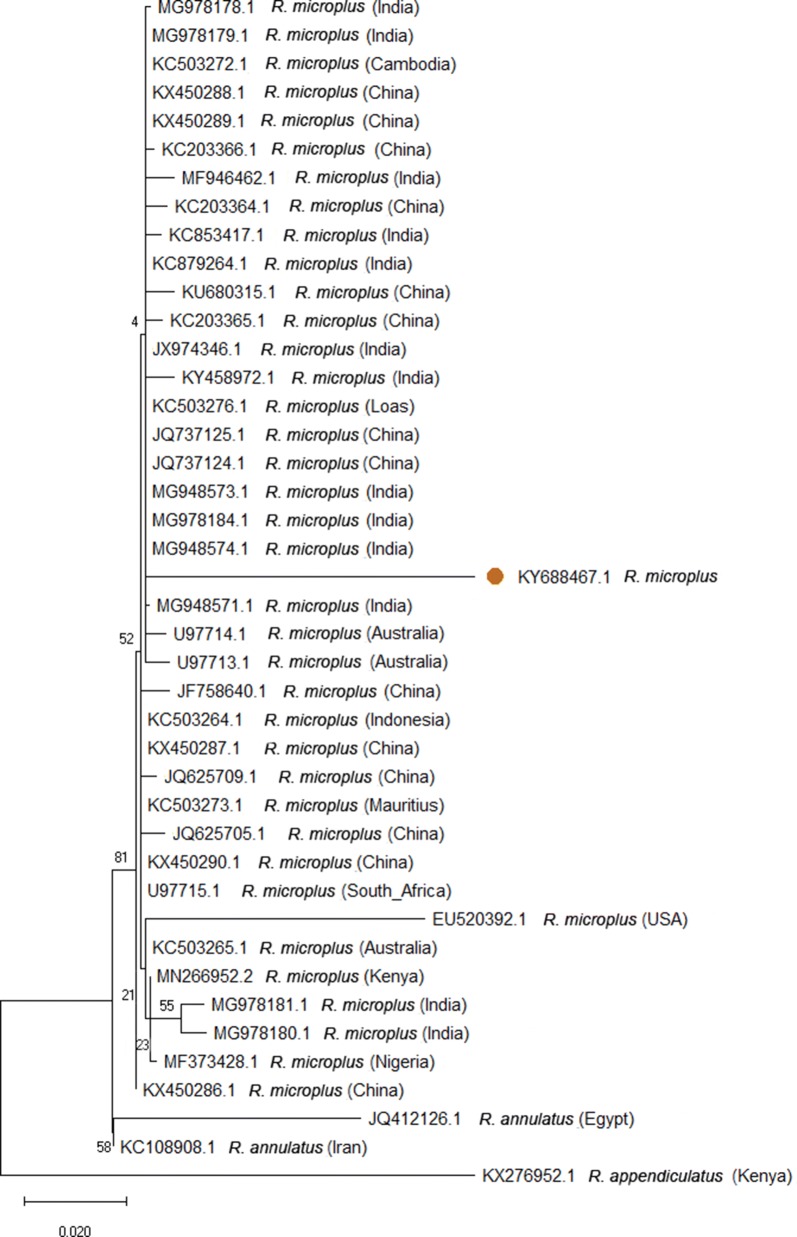
Phylogenetic analysis based on the ITS2 spacer of the ribosomal RNA gene cluster of ticks. A phylogenetic tree based on ITS2 rDNA sequences. The tree was generated by the maximum likelihood method based on the Tamura 3-parameter model. The analysis involved 32 nucleotide sequences. Orange circles represent samples sequenced in this study

Five of the 687 ticks that were identified as *R. microplus* using standard taxonomic keys were further analysed and all confirmed *R. microplus* by assessing sequence variation of their *12S* rRNA, *16S* rRNA and ITS2 regions. The genetic diversity of *R. microplus* ticks recovered from Uganda and those from elsewhere ranged between 0–0.075 (Table 2). Phylogenetic analysis of the *12S* rRNA (Fig. 3), *16S* rRNA (Fig. 4) and ITS2 (Fig. 5) regions revealed polymorphic sub-grouping with *R. microplus* collected from other parts of the world. The Ugandan *R. microplus* isolates were notably similar to those collected in Taiwan, Mozambique, Nigeria, the USA and South Africa.

**Table 2:** Estimates of evolutionary divergence using Ugandan *R. microplus 12S* rRNA, *16S* rRNA and ITS2 nucleotide sequences compared to *R. microplus* on GenBank

*R. microplus* sequences^a^	GenBank sequence ID	p*-*distance
*12S*		
KY688455	DQ003008.1 (Taiwan)	0.006
	EU921766.1 (Mozambique)	0.006
KY688459	DQ003008.1 (Taiwan)	0.000
	EU921766.1 (Mozambique)	0.000
*16S*		
KY688461	KY020993 (Brazil)	0.710
	EU918182.1 (South Africa)	0.663
	EU918187.1 (Mozambique)	0.071
ITS2		
KY688467	U97715.1 South Africa)	0.400
	MF373428.1 (Nigeria)	0.003
	MF373429.1 (Nigeria)	0.002
	EU520392.1 (USA)	0.038

^a^Present study

## Discussion

The high burden of adult ticks on cattle in Kadungulu sub-county, Serere District, south-eastern Uganda, confirms that ticks and associated diseases (anaplasmosis, babesiosis, theileriosis and heartwater) constitute a major constraint to livestock production in this region [25, 26]. It has been reported previously that *R. appendiculatus*, vector of *Theileria parva*, is the predominant tick species in Serere District [26–29]. Besides the discovery of *R. microplus* and the complete absence of *R. decoloratus*, the other tick species were the same as reported before in south-eastern Uganda [25–28]. However, the Ugandan tick population structure varies greatly between the different regions of the country, due to variation in microclimatic conditions [25–27]. For example, *Amblyomma lepidum*, *Hyalomma truncatum*, *Amblyomma gemma* and *Rhipicephalus pulchellus* thrive under the arid conditions of north-eastern Uganda [27, 30], and were therefore not found in this less arid study area.

In “The ixodid ticks of Uganda” Matthysse & Colbo [16] reported a systematic survey of ticks on livestock conducted between 1965–1966, wherein not a single *R. microplus* tick was found. Interestingly, before this survey, *R. microplus* was reported from Uganda by S. G. Wilson, who conducted a limited survey on cattle along the borders of Karamoja district, closer to the border with Kenya [17]. It is unlikely that *R. microplus* may have been missed during the nation-wide survey conducted by Matthysse & Colbo [16], now more than 50 years ago, although the sample size of 491 cattle was limited. Interestingly, our results clearly indicate that *R. microplus* has been overlooked for years, since it takes years to replace an indigenous population of *R. decoloratus* ticks [31]. Given the invasive nature of this tick species, exacerbated by poor animal movement control and communal grazing practices within the East African region, it may be the case that populations of *R. microplus* are now well established in Uganda.

Molecular phylogenetic analysis may be a useful tool to discern possible relationships between isolates collected from different geographical regions. In this study, the *12S* rRNA and ITS2 regions of the tick isolates from Uganda were identical to those previously isolated from Taiwan, Mozambique, Nigeria, USA and South Africa. It is therefore plausible that the *R. microplus* ticks collected from cattle in south-eastern Uganda were introduced on livestock imported from the southern parts of Africa. In the past 10–15 years, there have been significant importations of dairy cattle into Uganda from South Africa to improve the Ugandan dairy herd through cross-breeding [32]. However, a more extensive genotyping of ticks collected from different geographical areas is required to confirm this [26, 33].

Unprecedented levels of acaricide-resistant tick populations have recently been reported in Uganda [33]. The cause of this problem is due to farmer-related factors (acaricide overuse and misuse) potentiated by lack of national acaricide and animal movement control policies [33–35]. Under such favourable conditions, *R. microplus* tick populations are known to rapidly become acaricide-resistant as a result of target specific mutations and metabolic adaptations [36]. The introduction of *R. microplus* into Uganda is likely to exacerbate the already existing problem of ticks and tick-borne diseases in three ways. These include: (i) complete replacement of *R. decoloratus* by *R. microplus*, resulting in a national and probably regional upsurge of *R. microplus* populations; (ii) emergence of acaricide-resistant *R. microplus* populations; and (iii) a proportional increase of bovine babesiosis given that *R. microplus* is an efficient vector of *B. bovis* [11, 26, 32]. Unless effective national acaricide and animal movement control policies are instituted, the Ugandan livestock sector will suffer severe losses due to the direct effects of *R. microplus* infestation and bovine babesiosis.

## Conclusions

It was expected to find *R. decoloratus* among other tick species on cattle during a survey conducted in south-eastern Uganda. Instead, we discovered that *R. microplus* has completely displaced *R. decoloratus* in the six villages studied, an indigenous tick species previously known to this region. There is a need to determine the extent of spread of *R. microplus* throughout Uganda and to put in place effective control measures considering that *R. microplus* is capable of developing high levels of resistance towards the major classes of acaricides.

## Data Availability

Data supporting the conclusions of this article are included within the article. The newly generated sequences were submitted to the GenBank database under the accession numbers MK332390, MK332391, KY688455, KY688459, KY688461 and KY688467. The datasets used and/or analysed during the present study are available from the corresponding author upon reasonable request.

## Abbreviations

*12S* rRNA: *12S* ribosomal RNA gene
*16S* rRNA: *16S* ribosomal RNA gene
AAS: African Academy of Science
BMGF: Bill & Melinda Gates Foundation
DELTAS: Developing Excellence in Leadership, Training and Science
MUII: Makerere University-Uganda Virus Research Institute Centre of Excellence for Infection and Immunity Research and Training
MSA: multiple sequence alignment
NEPAD: New Partnership for Africa’s Development Planning and Coordinating Agency
DNA: deoxyribonucleic acid
RNA: ribonucleic acid
GPS: geographical positioning system
ITS 2: internal transcribed spacer 2
PBS: phosphate-buffered saline
